# A Decade of Ovarian Cancer in Indonesia: Epidemiology and Survival Analysis from 2010 to 2020

**DOI:** 10.3390/jcm14051692

**Published:** 2025-03-03

**Authors:** Primariadewi Rustamadji, Elvan Wiyarta, Kartiwa Hadi Nuryanto, Tricia Dewi Anggraeni, Fitriyadi Kusuma, Gatot Purwoto, Hariyono Winarto, Tantri Heliyanti, Hartono Tjahjadi, Amal Hayati, Ratu Ayu Dewi Sartika, Sabarinah Prasetyo, Andrijono Andrijono

**Affiliations:** 1 Department of Anatomic Pathology, Faculty of Medicine, Universitas Indonesia, Dr. Cipto Mangunkusumo National Hospital, Jakarta 10430, Indonesia; tantri.hellyanti@gmail.com (T.H.); htjahjadi@gmail.com (H.T.); amalarfiyan@gmail.com (A.H.); 2 Intensive Care Unit, University of Indonesia Hospital, Depok 16424, Indonesia; elvan.wiyarta@ui.ac.id; 3 Service Department, Risetku, Jakarta 12820, Indonesia; 4 Department Obstetrics and Gynecology, Faculty of Medicine, Universitas Indonesia, Dr. Cipto Mangunkusumo National Hospital, Jakarta 10430, Indonesiaanggi73@gmail.com (T.D.A.); gatotpurwoto@gmail.com (G.P.); hariyono.winarto@ui.ac.id (H.W.); andrijono@gmail.com (A.A.); 5 Faculty of Public Health, Universitas Indonesia, Depok 12345, Indonesia; ratuayu@ui.ac.id (R.A.D.S.); sabarinahprasetyo@gmail.com (S.P.)

**Keywords:** ovarian cancer, epidemiology, survival analysis, Indonesia, cancer database

## Abstract

**Background:** Ovarian cancer is a major global health issue, ranking among the foremost causes of cancer-related death in women. Despite its prevalence, epidemiology data and survival analysis pertinent to Indonesia are few. This study seeks to address the information gap by analyzing the demographic characteristics, clinical aspects, and survival outcomes of ovarian cancer patients in Indonesia from 2010 to 2020. **Methods:** This observational study utilized data from the Indonesian Cancer Database. This study included patients with a confirmed diagnosis of ovarian cancer. Data collected included age, parity, overall survival, geographic distribution, ethnicity, occupation, FIGO stage, tumor types, category, and degree of differentiation. Descriptive statistics were used to summarize the data, and Kaplan–Meier survival curves were employed to estimate survival probabilities over time. **Results:** The study cohort comprised 1065 patients with ovarian cancer. The cohort’s mean age was 52.41 (12.56) years, with 45.35% of patients residing in Jakarta. A majority were unemployed (75.77%) and of Javanese ethnicity (61.88%). Serous carcinoma (68.26%) was the most prevalent tumor types, while a high percentage of unknown FIGO stages (66.95%) limited staging data. The survival median time varied, with significant survival variation observed across tumor types, degrees of differentiation, and FIGO stages. Patients with serous carcinoma showed aggressive behavior with a median survival of 1 month, whereas clear cell carcinoma had a median survival of 9 months. **Conclusions:** This study highlights the need for improved early detection and equitable access to care to enhance survival outcomes for ovarian cancer patients in Indonesia.

## 1. Introduction

Ovarian cancer is a significant health concern worldwide, being one of the leading causes of cancer-related deaths among women [1]. Globally, ovarian cancer ranks as the eighth most common cancer among women, with more than 313,000 new cases reported in 2020 [1,2,3]. The five-year relative survival rate for ovarian cancer is about 50.8%, but this rate can vary significantly based on the stage at diagnosis and the types of ovarian cancer [4]. Ovarian cancer remains a significant public health concern in Asia, with 110,526 new cases recorded across Asian countries in 2012 [5]. The highest age-standardized incidence rates (ASR) were observed in Singapore (9.9 per 100,000), Kazakhstan (7.9 per 100,000), and Brunei (8.8 per 100,000) [5]. The survival rates for ovarian cancer in Asian countries vary widely, reflecting differences in healthcare access, early detection programs, and treatment availability. A meta-analysis by Maleki et al. [6] reported that the 1-year, 3-year, and 5-year survival rates of ovarian cancer in Asia were 73.65%, 61.31%, and 59.60%, respectively.

Despite the global prevalence of ovarian cancer, there is a significant gap in the epidemiological data and survival analysis specific to Southeast Asia, particularly Indonesia [5]. Most of the existing studies and data focus on Western populations, leaving a substantial knowledge gap regarding the demographic and clinical characteristics of ovarian cancer patients in this region [7]. Furthermore, Indonesia has unique healthcare challenges and demographic characteristics that can influence cancer diagnosis, treatment, and outcomes.

Ovarian cancer has a multifactorial etiology, influenced by genetic, hormonal, lifestyle, and environmental factors. BRCA1 and BRCA2 mutations, Lynch syndrome, and prolonged estrogen exposure increase the risk, as do obesity, smoking, and high-fat diets. Environmental exposures, such as asbestos and talc, have also been linked to ovarian carcinogenesis [5,6]. Despite these known risk factors, the disease remains difficult to detect early, emphasizing the need for improved screening and prevention strategies, particularly in Indonesia.

Previous studies on ovarian cancer in Indonesia are limited and often lack comprehensive data. A study on ovarian cancer patients in Jakarta revealed that most cases occurred in women aged 45–54, with a high prevalence of advanced-stage diagnoses and epithelial histotypes, highlighting the need for earlier detection and improved treatment strategies [7]. However, this study’s scope was restricted to a single center, limiting its generalizability to the broader Indonesian population [7]. Another study by Purbadi et al. found that advanced-stage ovarian cancer had poor survival rates, with cancer stage and adjuvant chemotherapy being significant factors affecting patient outcomes [8]. However, this study is also a single-center study with a limited sample size [8]. In fact, uniform data are needed to identify these hurdles, establish ovarian cancer control programs, and enhance treatment results [9].

To address this knowledge gap, an analysis of the uniform data was conducted on the Indonesian Cancer Database to better understand the distribution of ovarian cancer in the Indonesian population. The aim of this study is to analyze the demographic characteristics, clinical features, and survival outcomes of ovarian cancer patients in Indonesia over a decade. By addressing the knowledge gap in the epidemiology and survival of ovarian cancer in Indonesia, this study seeks to contribute to the global understanding of ovarian cancer and support the development of targeted interventions that can enhance patient care and survival outcomes in this region.

## 2. Methods

### 2.1. Study Design and Data Source

This study was a retrospective observational study utilizing data from the onco-gynecology division, department of obstetrics and gynecology, Cipto Mangunkusumo National Referral Hospital, which is a comprehensive Indonesian registry that collects detailed information on cancer cases across Indonesia. The database includes demographic data, clinical characteristics, treatment details, and follow-up information for patients diagnosed with various types of cancer. For this study, we extracted data specifically related to ovarian cancer diagnoses recorded between January 2010 and December 2020.

### 2.2. Study Population

The study population included patients with a confirmed diagnosis of ovarian cancer registered in the onco-gynecology division, department of obstetrics and gynecology, Cipto Mangunkusumo National Referral Hospital. The eligibility criteria for inclusion were patients with a confirmed diagnosis of ovarian cancer, complete demographic data (age, parity, province, ethnicity, and occupation), and clinical information (The International Federation of Gynecology and Obstetrics (FIGO) stage [10], category, tumor types, and degree of differentiation). Patients with incomplete demographic or clinical data were excluded from this study.

### 2.3. Variables and Data Collection

Data collected for each patient included age (recorded in years), parity (number of children), and overall survival (calculated as the time from diagnosis to death or last follow-up, recorded in months). Geographic distribution was determined by the province of residence, categorized into DKI Jakarta, Jawa Barat, Banten, and other provinces. Ethnicity was recorded as Jawa, Sunda, Betawi, and other ethnic groups. Occupation was categorized as employed or unemployed. The FIGO stage was used to classify the extent of ovarian cancer. Tumor types were classified histologically according to the 2020 WHO classification of ovarian tumors [11], and categories were broadly categorized into clear cell tumors, endometrioid tumors, germ cell tumors, mucinous tumors, pure sex cord tumors, serous tumors, and other carcinomas. However, the database used in this study did not consistently differentiate between high-grade and low-grade serous carcinoma, limiting our ability to analyze these subtypes separately. The degree of differentiation was recorded as Grade 1, Grade 2, Grade 3, or unknown.

### 2.4. Statistical Analysis

Descriptive statistics were utilized to summarize the demographic and clinical characteristics of the patients. Continuous variables were reported as means and standard deviations (SD), while categorical variables were presented as frequencies and percentages. Kaplan–Meier survival curves were employed to estimate survival probabilities over time [12], stratified by tumor types, category, and degree of differentiation. Median survival times were calculated for each category, and the number of patients at risk at various time points was recorded. The statistical software used for the analysis included R version 4.4.3, utilizing packages such as lubridate, ggsurvfit, gtsummary, tidycmprsk, knitr, dplyr, survival, ggplot2, here, and tibble.

### 2.5. Ethical Considerations

This study adhered to the Strengthening the Reporting of Observational Studies in Epidemiology (STROBE) guidelines to ensure comprehensive and transparent reporting [13]. This study’s design, data collection, analysis, and reporting were conducted following these guidelines to enhance the reliability and reproducibility of the findings. This study was conducted in accordance with the Declaration of Helsinki [14]. Approval was obtained from the institutional review board of the University of Indonesia in March 2024 (ethics approval number Ket-121/UN2.F10.D11/PPM.00.02/2024). All patient data were anonymized to ensure confidentiality.

## 3. Result

The study cohort comprised 1065 patients with ovarian cancer, which can be seen in Table 1. The mean age was 52.41 years (SD = 12.56), and the mean parity was 1.64 children (SD = 1.05). The mean overall survival was 5.07 months (SD = 6.50). This finding aligns with the predominantly advanced-stage presentation observed in our cohort. The high percentage of unknown FIGO stages (66.95%) and the predominance of Stage IIIC cases further suggest that delayed diagnosis significantly impacts survival. The majority of patients were from DKI Jakarta (45.35%), followed by Jawa Barat (36.96%). In terms of ethnicity, the largest group was Jawa (61.88%), followed by Sunda (14.84%). A significant proportion of patients were unemployed (75.77%).

The FIGO staging showed a high percentage of unknown stages (66.95%), with Stage IIIC being the most commonly known stage (10.52%). The most frequent tumor types were serous carcinomas (68.26%), followed by clear cell carcinomas (8.17%) and mucinous carcinomas (6.67%). Other carcinomas were predominant (68.26%). However, due to database limitations, we were unable to differentiate between high-grade and low-grade subtypes. This distinction is clinically relevant, as high-grade serous carcinoma (HGSC) is more aggressive and less responsive to chemotherapy than low-grade serous carcinoma (LGSC). The lack of differentiation in our study highlights the need for improved histopathological data collection in Indonesian cancer registries to refine prognostic assessments and therapeutic strategies. The degree of differentiation was unknown for most patients (79.44%), but among those known, Grade 3 was the most common (8.45%).

**Figure 1:**
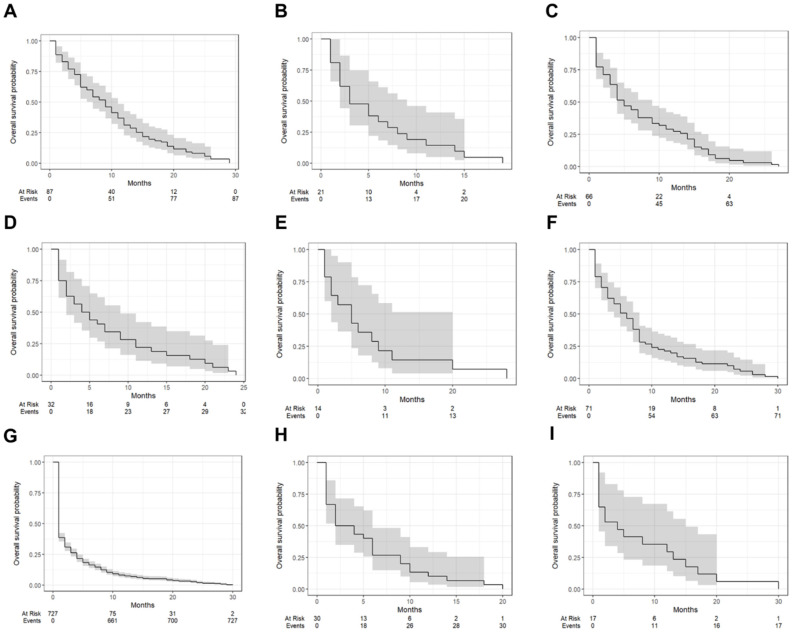
Kaplan–Meier survival curves depicting overall survival outcomes for patients with various tumor types, including clear cell carcinoma (**A**), dysgerminoma (**B**), endometrioid carcinoma (**C**), adult granulosa cell tumor (**D**), immature teratoma (**E**), mucinous carcinoma (**F**), serous carcinoma (**G**), undifferentiated and dedifferentiated carcinomas (**H**), and yolk sac tumor (**I**). Each curve represents survival probability over time, with shaded areas indicating confidence intervals.

Figure 1 illustrates the Kaplan–Meier survival curves stratified by tumor types, while Table 2 summarizes the one-year survival probabilities and median survival times for each tumor type. Clear cell carcinoma demonstrated the highest one-year survival rate at 31.0% (95% CI: 22.7–42.5%) with a median survival time of 9 months (IQR: 7–11). In contrast, serous carcinoma exhibited the poorest prognosis, with a one-year survival rate of 7.4% (95% CI: 5.6–9.6%) and a median survival time of only 1 month (IQR: 1–2). Dysgerminoma and yolk sac tumors showed intermediate outcomes, with one-year survival rates of 14.3% (95% CI: 5.0–40.7%) and 29.4% (95% CI: 14.2–61.4%), respectively, and median survival times of 3 months (IQR: 2–9) and 4 months (IQR: 1–15). Endometrioid carcinoma had a one-year survival rate of 27.3% (95% CI: 18.4–40.4%) and a median survival time of 5 months (IQR: 4–9). Other tumor types, including mucinous carcinoma (21.1%, median survival: 6 months), undifferentiated/dedifferentiated carcinomas (10.0%, median survival: 3 months), and adult granulosa cell tumors (21.9%, median survival: 4.5 months), reflected varying survival outcomes with overlapping confidence intervals. Immature teratomas displayed considerable variability with a median survival time of 5 months (IQR: 2–20) and a one-year survival rate of 14.3%.

**Figure 2:**
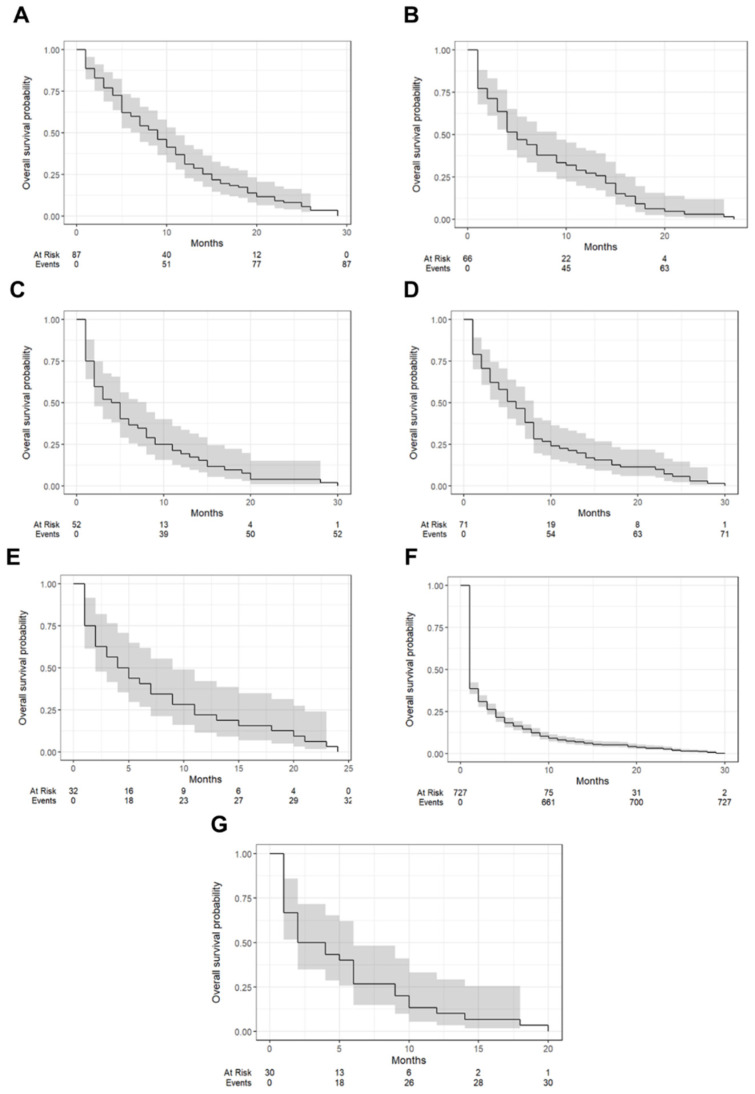
Kaplan–Meier survival curves illustrating overall survival outcomes for patients categorized by tumor types. Categories include: clear cell tumors (**A**), endometrioid tumors (**B**), germ cell tumors (**C**), mucinous tumors (**D**), pure sex cord tumors (**E**), serous tumors (**F**), and other carcinomas (**G**). Each curve represents survival probability over time, with shaded areas denoting confidence intervals.

Figure 2 presents Kaplan–Meier survival curves grouped by tumor categories, while Table 3 outlines the one-year survival rates and median survival times for each category. Clear cell tumors demonstrated the best prognosis, with a one-year survival rate of 31.0% (95% CI: 22.7–42.5%) and a median survival time of 9 months (interquartile range [IQR]: 7–11). Endometrioid tumors followed with a one-year survival rate of 27.3% (95% CI: 18.4–40.4%) and a median survival time of 5 months (IQR: 4–9). Serous tumors were associated with the poorest outcomes, showing a one-year survival rate of 7.4% (95% CI: 5.8–9.6%) and a median survival time of just 1 month (IQR: 1–2). Mucinous tumors and pure sex cord tumors demonstrated similar survival profiles, with one-year survival rates of 21.1% (95% CI: 13.5–33.1%) and 21.9% (95% CI: 11.4–42.1%), respectively, and median survival times of 6 months (IQR: 4–8) and 4.5 months (IQR: 2–9). Germ cell tumors showed a one-year survival rate of 19.2% (95% CI: 1.10–33.6%) and a median survival time of 4.5 months (IQR: 2–8), while other carcinomas had a one-year survival rate of 10.0% (95% CI: 3.4–29.3%) and a median survival time of 3 months (IQR: 2–6).

**Figure 3:**
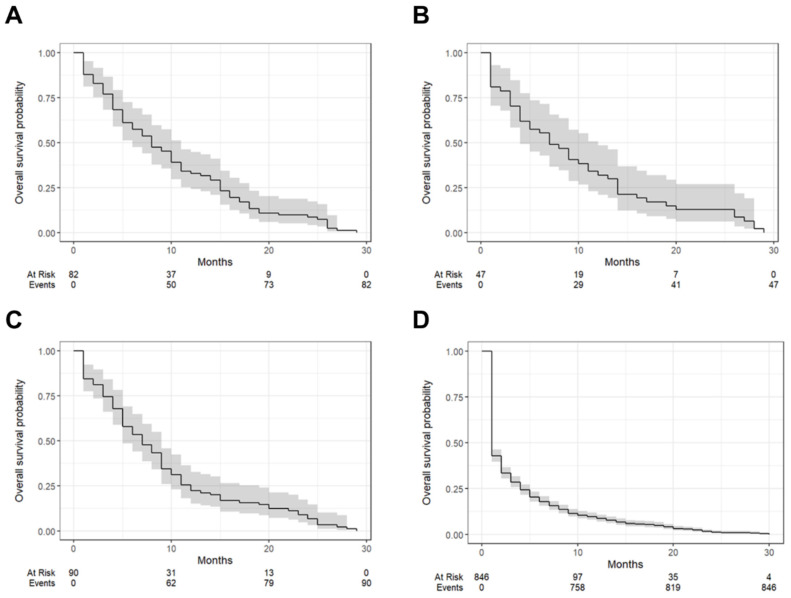
Kaplan–Meier survival curves illustrating overall survival outcomes based on the degree of tumor differentiation. Groups include Grade 1 (**A**), Grade 2 (**B**), Grade 3 (**C**), and Unknown grade (**D**). Each curve represents survival probability over time, with shaded areas indicating confidence intervals.

Figure 3 illustrates Kaplan–Meier survival curves based on the degree of tumor differentiation, and Table 4 summarizes the one-year survival rates and median survival times for each grade. Grade 1 tumors exhibited the most favorable outcomes, with a one-year survival rate of 32.9% (95% CI: 5.2–44.8%) and a median survival time of 8 months (IQR: 6–11). Grade 2 tumors followed closely, with a one-year survival rate of 31.9% (95% CI: 21.0–48.5%) and a median survival time of 7 months (IQR: 4–12). Tumors classified as Grade 3 showed slightly poorer outcomes, with a one-year survival rate of 22.2% (95% CI: 15.1–32.7%) and a median survival time of 7 months (IQR: 5–9). Cases with unknown differentiation had the worst prognosis, with a one-year survival rate of only 8.51% (95% CI: 6.82–10.6%) and a median survival time of 1 month (IQR: 1–2).

## 4. Discussion

This study provides a comprehensive analysis of the demographic characteristics, clinical features, and survival outcomes of ovarian cancer patients in Indonesia over a decade, from 2010 to 2020, using data from the onco-gynecology division, department of obstetrics and gynecology, Cipto Mangunkusumo National Referral Hospital. The findings highlight significant insights and identify critical areas for improvement in the management of ovarian cancer in Indonesia.

The overall survival of patients in this cohort was relatively poor, with a median survival of 5.07 months. This is significantly lower than the 5-year survival rates reported in many Asian–Pacific countries. According to Maleki et al. [6], the average 5-year survival rate for ovarian cancer in Asia is 59.60%, with some countries reporting rates above 80%. Specifically, Turkey has the highest 5-year survival (85.27%), followed by Japan (55%) and South Korea (60%), while India reports the lowest survival rate (46.72%). Our findings indicate that Indonesia’s survival outcomes are substantially worse than the regional average, likely due to late-stage diagnosis (66.95% of cases with unknown FIGO staging or presenting at Stage IIIC), limited access to specialized oncology care, and suboptimal chemotherapy utilization.

When comparing Indonesia’s survival rates to high-income countries, the disparities become even more pronounced. For instance, in Australia, the 5-year survival rate for ovarian cancer is 46–50%, while in the United States and Canada, it exceeds 53% [6]. These differences emphasize the need for improved early detection programs, increased public awareness, and better access to advanced treatment modalities. Additionally, Razi et al. [5] highlighted the correlation between ovarian cancer incidence and the Human Development Index (HDI), where countries with higher HDI scores tend to have higher incidence rates due to better detection but also improved survival due to superior healthcare systems. Indonesia’s relatively poor HDI ranking and healthcare disparities may further contribute to the lower survival rates observed in our study.

This study also underscores substantial variations in survival rates among tumor types. Clear cell carcinoma had the best prognosis, with a one-year survival rate of 31.0% and a median survival time of 9 months, while serous carcinoma exhibited the worst outcomes (one-year survival rate of 7.4%, median survival of 1 month). These findings align with global literature indicating the aggressive nature and chemoresistance of serous carcinoma [15,16,17]. On the other hand, clear cell carcinoma’s relatively better prognosis may be linked to its distinct molecular profile, characterized by frequent ARID1A and PIK3CA mutations, which could offer actionable therapeutic targets [18,19]. Germ cell tumors and sex cord–stromal tumors showed intermediate survival outcomes, likely reflecting their typically earlier presentation and higher chemosensitivity [20]. The variability observed in immature teratomas suggests that age at diagnosis, tumor stage, and histological grading may substantially influence prognosis [21]. Comparative studies from other Asian countries reveal similar trends [6]. This variation can be attributed to differences in healthcare infrastructure, availability of specialized care, and socioeconomic factors.

The high proportion of unknown FIGO stages (66.95%) highlights a critical gap in the diagnostic workup of ovarian cancer in this cohort. Among the known stages, Stage IIIC was most common, reflecting late-stage presentation as a significant challenge in ovarian cancer management in Indonesia. Early detection is crucial, as studies have shown that early-stage diagnosis is associated with a markedly higher survival rate. For example, a study in Canada reported a five-year survival rate of 62.0% for early-stage epithelial ovarian cancer [22]. This highlights the importance of effective screening programs and public awareness to facilitate early diagnosis. This trend is consistent with reports from other low- and middle-income countries, where late diagnosis results from limited access to diagnostic tools, lack of public awareness, and inadequate screening programs [9,23].

The high incidence of advanced-stage patients identified in our investigation indicates significant deficiencies in the existing referral and early detection systems. This discovery highlights the necessity for immediate policy enhancements to mitigate referral delays and improve early detection capability. Timely diagnosis is crucial for enhancing treatment efficacy and survival rates in patients; therefore, prioritizing the optimization of policies to minimize delays is essential [24]. Comparative investigations with other nations indicate that analogous delays in cancer diagnosis may also transpire abroad, prompting inquiries into whether these patterns primarily result from the inherent problems of cancer detection or from systemic deficiencies within certain healthcare systems [25,26]. This knowledge is especially significant for guiding policy modifications, as comprehending the relative impact of these components may facilitate the development of more focused actions [9,23].

A proactive strategy is required to overcome these gaps, emphasizing increased sensitivity to patient-reported symptoms. Identifying certain symptoms linked to advanced phases and selecting them for additional examination may facilitate improved early diagnosis [9]. Studies demonstrate that the application of systematic criteria for recognizing and prioritizing certain symptoms can enhance diagnostic precision and diminish the likelihood of missing instances that could be detected sooner [27]. In addition to this method, efforts to document prevalent signs and symptoms associated with advanced-stage presentations should enhance early detection rates, as practitioners become more aware of these markers during routine assessments [9].

The limitations of this study include its retrospective design and reliance on hospital-based records, which differ from population-based cancer registries commonly used for broader epidemiological studies. While hospital-based records provide valuable clinical insights, they may not fully capture cases managed in different healthcare settings. This can lead to variations in case representation, particularly in relation to disease stage at diagnosis and treatment accessibility. The high percentage of unknown FIGO stages and degrees of differentiation may also affect the generalizability of the findings. While survival analysis often incorporates statistical comparisons such as the log-rank test, descriptive data presentation is equally essential in capturing clinically relevant trends. In this study, survival outcomes were presented descriptively rather than through statistical comparison due to the presence of selection bias and unbalanced group sizes, which could compromise the reliability of comparative survival analysis. Given these limitations, this study emphasizes the importance of enhancing survival data collection and analysis methodologies. Future research should focus on balanced cohort designs and refined statistical approaches to enable more accurate survival comparisons. In terms of methodology, instead of reporting 95% CIs for the median survival time, this study presents IQR to describe variability. The IQR was chosen over CIs because of censoring and sample size limitations, which could reduce the reliability of CI estimates. Despite these limitations, this study provides valuable insights into the epidemiology and survival of ovarian cancer in Indonesia and highlights critical areas for intervention.

## 5. Conclusions

In conclusion, this study underscores the urgent need for improved early detection, equitable access to specialized care, and adherence to treatment guidelines to enhance survival outcomes for ovarian cancer patients in Indonesia. By addressing these challenges, it is possible to reduce the burden of ovarian cancer and improve the quality of life for affected women in Indonesia. Further research should focus on prospective studies and interventions to address the identified gaps and improve patient outcomes.

## Figures and Tables

**Table 1:** The descriptive statistics of all included patients.

Variables	Descriptive Statistics
Age, years [mean (SD)]	52.41 (12.56)
Parity, children [mean (SD)]	1.64 (1.05)
Overall Survival, months [mean (SD)]	5.07 (6.50)
Province, N (%)	
DKI Jakarta	483 (45.35%)
Jawa Barat	383 (35.96%)
Banten	79 (7.42%)
Other	120 (11.27%)
Ethnic, N (%)	
Jawa	695 (61.88%)
Sunda	158 (14.84%)
Betawi	100 (9.39%)
Other	148 (13.90%)
Occupation, N (%)	
Unemployed	807 (75.77%)
Employed	258 (24.23%)
FIGO stage, N (%)	
Stage IA	49 (4.60%)
Stage IB	13 (1.22%)
Stage IC1	46 (4.32%)
Stage IC2	2 (0.19%)
Stage IC3	8 (0.75%)
Stage IIA	8 (0.75%)
Stage IIB	17 (1.60%)
Stage IIIA	18 (1.69%)
Stage IIIA1	5 (0.47%)
Stage IIIA2	3 (0.28%)
Stage IIIB	18 (1.69%)
Stage IIIC	112 (10.52%)
Stage IVA	38 (3.57%)
Stage IVB	15 (1.41%)
Unknown	713 (66.95%)
Tumor Types, N (%)	
clear cell carcinoma	87 (8.17%)
dysgerminoma	21 (1.97%)
endometrioid carcinoma	66 (6.20%)
adult granulosa cell tumor	32 (3.00%)
immature teratoma	14 (1.31%)
mucinous carcinoma	71 (6.67%)
serous carcinoma	727 (68.26%)
undifferentiated and dedifferentiated carcinomas	30 (2.82%)
yolk sac tumor	17 (1.60%)
Category, N (%)	
clear cell tumors	87 (8.17%)
endometrioid tumors	66 (6.20%)
germ cell tumors	52 (4.88%)
mucinous tumors	71 (6.67%)
pure sex cord tumors	30 (2.82%)
serous tumors	32 (3.00%)
other carcinomas	727 (68.26%)
Degree Differentiation, N (%)	
Grade 1	82 (7.70%)
Grade 2	47 (4.41%)
Grade 3	90 (8.45%)
Unknown	846 (79.44%)

**Table 2:** One-year survival and median survival time based on tumor types.

Tumor Types	One-Year Survival (95% CI)	Median Survival Time (Months [IQR])
clear cell carcinoma	31.0% (22.7–42.5%)	9 (7–11)
dysgerminoma	14.3% (5.0–40.7%)	3 (2–9)
endometrioid carcinoma	27.3% (18.4–40.4%)	5 (4–9)
adult granulosa cell tumor	21.9% (11.4–42.1%)	4.5 (2–9)
immature teratoma	14.3% (39.6–51.5%)	5 (2–20)
mucinous carcinoma	21.1% (13.5–33.1%)	6 (4–8)
serous carcinoma	7.4% (5.6–9.6%)	1 (1–2)
undifferentiated and dedifferentiated carcinomas	10.0% (3.4–29.3%)	3 (2–6)
yolk sac tumor	29.4% (14.2–61.4%)	4 (1–15)

**Table 3:** One-year survival and median survival time based on categories.

Tumor Types	One-Year Survival (95% CI)	Median Survival Time (Months [IQR])
clear cell tumors	31.0% (22.7–42.5%)	9 (7–11)
endometrioid tumors	27.3% (18.4–40.4%)	5 (4–9)
germ cell tumors	19.2% (1.10–33.6%)	4.5 (2–8)
mucinous tumors	21.1% (13.5–33.1%)	6 (4–8)
pure sex cord tumors	21.9% (11.4–42.1%)	4.5 (2–9)
serous tumors	7.4% (5.8–9.6%)	1 (1–2)
other carcinomas	10.0% (3.4–29.3%)	3 (2–6)

**Table 4:** One-year survival and median survival time based on degree of differentiation.

Tumor Types	One-Year Survival (95% CI)	Median Survival Time (Months [IQR])
Grade 1	32.9% (5.2–44.8%)	8 (6–11)
Grade 2	31.9% (21.0–48.5%)	7 (4–12)
Grade 3	22.2% (15.1–32.7%)	7 (5–9)
Unknown	8.51% (6.82–10.6%)	1 (1–2)

## Data Availability

All data generated or analyzed during this study are included in this published article.
